# Projected soil organic carbon loss in response to climate warming and soil water content in a loess watershed

**DOI:** 10.1186/s13021-021-00187-2

**Published:** 2021-08-16

**Authors:** Fubo Zhao, Yiping Wu, Jinyu Hui, Bellie Sivakumar, Xianyong Meng, Shuguang Liu

**Affiliations:** 1 Department of Earth & Environmental Science, School of Human Settlements and Civil Engineering, Xi’an Jiaotong University, Xi’an, 710049 Shaanxi China; 2 Department of Civil Engineering, Indian Institute of Technology Bombay, Powai, Mumbai, Maharashtra 400076 India; 3 College of Resources and Environmental Sciences, China Agricultural University, Beijing, 100094 China; 4 National Engineering Laboratory for Applied Technology of Forestry and Ecology in South China, Central South University of Forestry and Technology, Changsha, 410004 China

**Keywords:** Carbon cycle, Climate change, Soil organic carbon, Soil water content, SWAT-DayCent

## Abstract

**Background:**

Soil organic carbon (SOC) plays a crucial role in the global carbon cycle and terrestrial ecosystem functions. It is widely known that climate change and soil water content (SWC) could influence the SOC dynamics; however, there are still debates about how climate change, especially climate warming, and SWC impact SOC. We investigated the spatiotemporal changes in SOC and its responses to climate warming and root-zone SWC change using the coupled hydro-biogeochemical model (SWAT-DayCent) and climate scenarios data derived under the three Representative Concentration Pathways (RCPs2.6, 4.5, and 8.5) from five downscaled Global Climate Models (GCMs) in a typical loess watershed––the Jinghe River Basin (JRB) on the Chinese Loess Plateau.

**Results:**

The air temperature would increase significantly during the future period (2017–2099), while the annual precipitation would increase by 2.0–13.1% relative to the baseline period (1976–2016), indicating a warmer and wetter future in the JRB. Driven by the precipitation variation, the root-zone SWC would also increase (by up to 27.9% relative to the baseline under RCP4.5); however, the SOC was projected to decrease significantly under the future warming climate. The combined effects of climate warming and SWC change could more reasonably explain the SOC loss, and this formed hump-shaped response surfaces between SOC loss and warming-SWC interactions under both RCP2.6 and 8.5, which can help explain diverse warming effects on SOC with changing SWC.

**Conclusions:**

The study showed a significant potential carbon source under the future warmer and wetter climate in the JRB, and the SOC loss was largely controlled by future climate warming and the root-zone SWC as well. The hump-shaped responses of the SOC loss to climate warming and SWC change demonstrated that the SWC could mediate the warming effects on SOC loss, but this mediation largely depended on the SWC changing magnitude (drier or wetter soil conditions). This mediation mechanism about the effect of SWC on SOC would be valuable for enhancing soil carbon sequestration in a warming climate on the Loess Plateau.

**Supplementary Information:**

The online version contains supplementary material available at 10.1186/s13021-021-00187-2.

## Background

Soil is the largest carbon (C) reservoir and stores about 1505 Pg C in the top one meter, approximately twice as much the amount in the atmosphere or three times in the terrestrial vegetation [1–3]. Soil organic carbon (SOC) forms the majority of the terrestrial soil C pool (accounting for nearly 62% of the soil carbon pool) and plays an important role in the global C cycle and balance [4–6]. Even a small change in the SOC can substantially affect not only the climate but also the stability of ecosystems, because of its decisive role in the exchange of carbon between the soil and atmosphere and plant growth/food production [7–10]. Therefore, understanding the spatiotemporal changes of SOC and the associated driving factors is of critical importance to evaluate the feedbacks between terrestrial C cycle and climate change and the maintenance of the ecosystem functions [11–13].

Whether soil C pool acts either as a source or as a sink for atmospheric CO_2_ is largely controlled by the changes in climate and soil water content (SWC) [14–20]. Climate change, especially climate warming, can directly or indirectly impact the SOC decomposition through controlling the soil microbes, enzyme activities, and soil respiration [21]. During the past several decades, although many studies have examined the effects of climate warming on the SOC dynamics, there are still debates on this issue due to the contradictory results reported [3, 22]. Some modeling studies and meta-analysis showed that climate warming could stimulate the loss of soil C into the atmosphere because of the stronger warming effects on respiration than photosynthesis, leading to positive land C-climate feedback [23–26]. For example, through compiling data from the published literature, Crowther et al. [7] found that climate warming could remarkably reduce the SOC stocks and the warming effects were largely contingent on the size of the initial SOC stocks. Using a biogeochemical model, Zhao et al. [11] found that SOC would significantly decrease under a warming climate in a typical loess hilly and gully watershed. In contrast, results from some experimental studies showed that warming could also stimulate the carbon uptake in some ecosystems, leading to negative C-climate feedbacks [27–29]. For example, Zhang et al. [28] found that the temperature rise might enhance the CO_2_ sink in both boreal and temperate ecosystems. Some global-scale studies have also revealed that the warming effects on the SOC decomposition might be overestimated because the SOC decomposition rates were remarkably constant across the mean annual temperature gradient [29]. These contradictory results suggest that the relationship between climate warming and the SOC dynamics and the C-climate feedbacks remain uncertain, thus constraining the accurate prediction of the future climate change.

Soil water content is another important factor driving the SOC dynamics. It is generally known that SWC plays a crucial role in vegetation growth and C substrate supply for microbial activities under different climate conditions [30, 31]. High SWC may stimulate the C uptake (e.g., the ecosystem productivity) or release it (e.g., the soil respiration) in a warm climate, but excessive SWC could depress them in relatively wet climate conditions [32–34]. For example, in cool tropical forests, high water availability can slow the C cycling, while in warm tropical forests it can enhance the ecosystem productivity and SOC decomposition [32]. In the arid and semi-arid steppes, high SWC can accelerate the soil respiration rate, leading to a significant C loss from soil [35]. The SWC also plays a crucial role in controlling the responses of C cycle to climate warming [36]. The warming effects on C would flip from positive to negative as the soil changes from wet to dry, demonstrating the key role of SWC on warming-induced C gain or loss [37]. A recent study revealed that warming could stimulate net carbon uptake under wet conditions but depress it under very dry conditions [27]. These findings suggest that the C cycle is closely linked with SWC, and this relationship could be more complex under climate warming. Furthermore, in the context of climate change, shifts in soil water caused by precipitation will continuously exert uncertain influences on the future C dynamics. Moreover, it is still not clear how climate warming and SWC interact with each other to impact the SOC dynamics at watershed or regional scale, which largely hampers the modeling and projections of the global C cycling.

The overall goal of this study was to investigate the spatiotemporal changes in SOC and its responses to future changes in climate and SWC in a loess watershed. To this end, the Jinghe River Basin (JRB), a large typical loess hilly-gully watershed on the Loess Plateau, was studied. A coupled hydro-biogeochemical model—SWAT-DayCent—combined with projections from five downscaled Global Climate Models (GCMs) (GFDL-ESM2M, HadGEM2-ES, IPSL-CM5A-LR, MIROC-ESM-CHEM, and NoerESM1-M) under three Representative Concentration Pathways (RCPs) (RCP2.6, RCP4.5, and RCP8.5) was used. Specifically, we aimed to: (1) investigate the spatiotempral changes in SOC and SWC under current (1976–2016) and projected future climate scenarios (2017–2099), and (2) examine the responses of SOC to both climate warming and SWC change in the JRB.

## Methods

### Study area

**Fig. 1:**
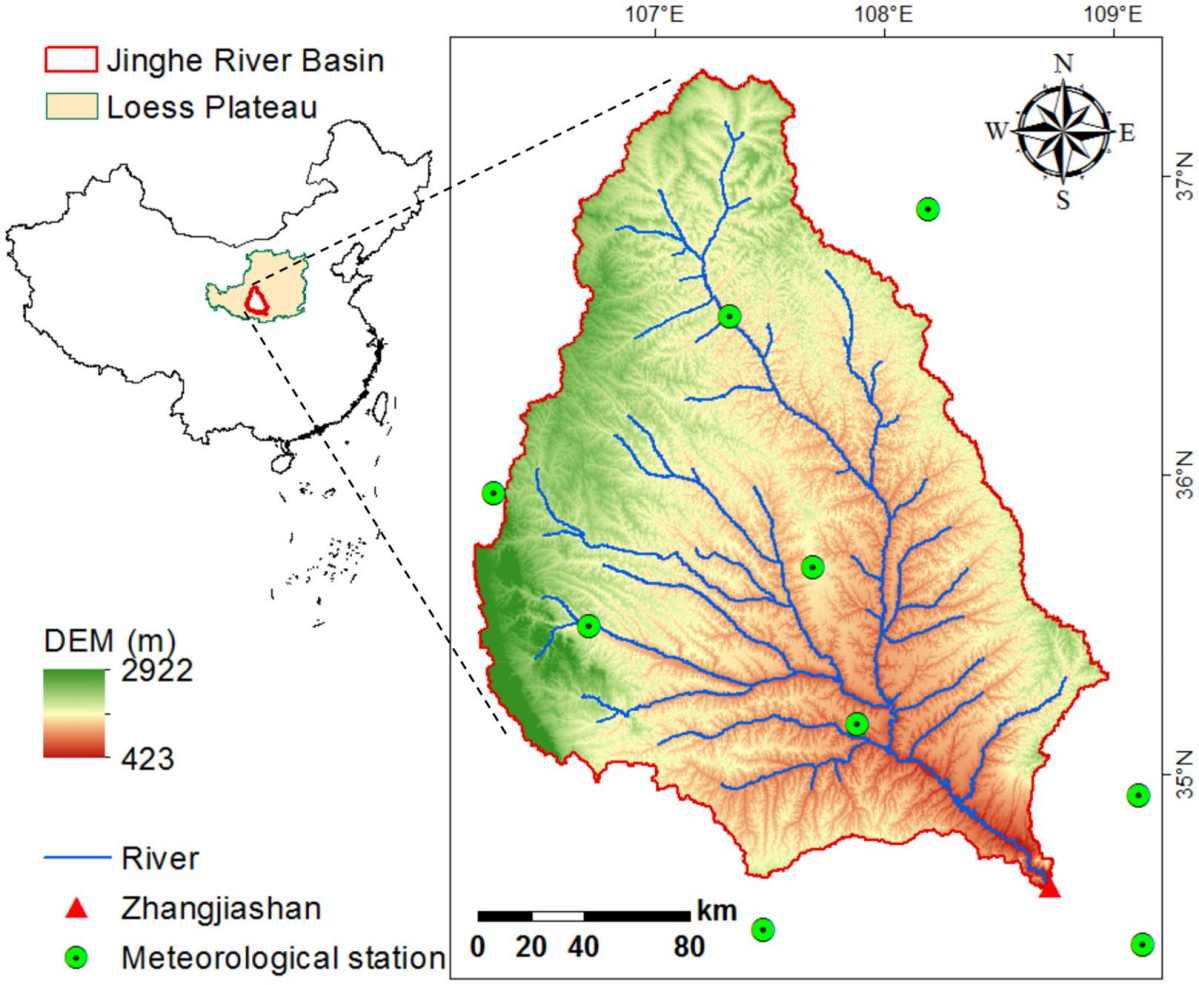
Location and DEM of the Jinghe River Basin. The red triangle indicates the Zhangjiashan Gaging station and the green circles indicate the meteorological stations

The Jinghe River Basin (106°–108° E, 34°–37° N) is a typical loess hilly-gully watershed located in the southern part of the Chinese Loess Plateau (Fig. 1). The Jinghe River originates from the Liupan Mountain and has a total length of 455 km [14]. The watershed lies in the semi-humid and semi-arid transitional zone with a typical continental climate [38]. The annual average precipitation and temperature are about 350–600 mm and 8–13 °C (from north to south), respectively, and approximately 80% of precipitation occurs in the flood season between July and September. Grassland, cropland, and forest are the major land use types in the basin, accounting for 90% of the total area [39]. The watershed is often subject to severe water shortage and soil erosion due to the dry climate and loosened land surface.

### Description of SWAT-DayCent

The SWAT-DayCent model, coupled from the widely-used watershed-scale distributed hydrological model (Soil and Water Assessment Tool, SWAT) [40] and the principle biogeochemical model (daily time step version of the CENTURY model, DayCent) [41, 42], was developed by Wu et al. [43]. It can simultaneously simulate the hydrological and biogeochemical processes at the watershed scale. In the coupled running process, the SWAT was set as the basic framework and the DayCent was embedded into SWAT at the HRU (Hydrologic Response Unit) level, together with some new functions for information transfer and format conversion. For example, some functions were used to change the soil structure to meet DayCent requirement and transform the soil property information in each HRU into DayCent. The major watershed-scale outputs of SWAT-DayCent include hydrological components (e.g., streamflow, soil water, ET) produced by SWAT and biogeochemical components (e.g., NPP (Net Primary Productivity), SOC, soil respiration) produced by DayCent, enabling users to integrate the modeling of water and carbon at the same spatiotemporal input and at the same computing units, facilitating the analysis of interaction between water and carbon at the watershed scale. In DayCent simulations, the SOC model simulates SOC dynamics for three SOC pools–soil active, slow, and passive pools. The active pool includes soil microbes and microbial products with short turnover times (1–3 months), the slow SOC pool includes resistant plant material that have turnover times ranging from 10 to 50 years depending on climate, and the passive pool includes physically and chemically stabilized SOC that is very resistant to decomposition [44]. The decomposition of SOC is microbially-mediated with an associated microbial respiration CO_2_ loss. Each SOC pool has specific maximum decomposition rates with maximum being reduced by an abiotic soil decomposition factor that is controlled by the soil moisture and soil temperature. In addition, the SOC decomposition is also largely controlled by soil texture. For example, the net effects of the soil texture on active and slow SOC decomposition is to increase soil carbon stabilization for soils with low sand content and high clay content. Extensive details of the development and applications of SWAT-DayCent can be found in our previous studies [11, 12, 43, 45].

### Model input and verification

The ArcSWAT (version 2012) was employed in this study to automate the input parameters. The required inputs for SWAT include topography, land use, soil type, and meteorological information. The digital elevation model (DEM) was obtained from the Shuttle Radar Topography Mission (SRTM) with a 90-m resolution to delineate the watershed and define the stream networks. The land use and soil property data were obtained from the Ecological and Environmental Science Data Center for West China with a 1-km resolution. The historical daily meteorological data, including precipitation, maximum and minimum temperature, relative humidity, wind speed, and sunshine duration, were obtained from the data center of China Meteorological Administration (CMA), covering the study period from 1976 to 2016. The sunshine duration here was used to calculate the solar radiation required for SWAT [39].

As in our previous studies [12, 14, 45], the SWAT-DayCent was calibrated and validated for performance in hydrological and carbon cycle simulations by using the monthly streamflow from the Zhangjiashan station (see its location in Fig. 1) and remotely-sensed NPP. The use of multiple criteria performance evaluation measures, including NSE (Nash–Sutcliffe Efficiency), R^2^ (correlation coefficient), PB (percentage bias), and RMSE (root mean square error), showed that the SWAT and DayCent could simulate the water and carbon cycles with satisfactory performance [46]. Details of the calibration and validation schemes and the model performance can be found in our previous publications [12, 14, 45].

### Future climate datasets

In this study, we used the future climate datasets (precipitation and maximum/ minimum air temperature) that were downscaled from five GCMs (GFDL-ESM2M, HadGEM2-ES, IPSL-CM5A-LR, MIROC-ESM-CHEM, and NoerESM1-M (Table 1)) by the Inter-Sectoral Impact Model Inter-comparison Project (ISI-MIP) under Representative Concentration Pathway (RCP) 2.6 (low emission pathway), RCP4.5 (low-to-moderate emission pathway), and RCP8.5 (high emission pathway) [47, 48]. Unlike the Coupled Model Inter-comparison Project Phase 5 (CMIP5), the data sets from ISI-MIP were bias-corrected by comparing with the Climatic Research Unit (CRU) data and downscaled to 0.5° × 0.5° spatial resolution [49] (Table 1). We verified these data sets against historical data in our previous study, and showed that the ISI-MIP data could largely reflect the real climatic conditions of the JRB [11]. With this, the future climate forcing data was used to drive the SWAT-DayCent model to predict the hydro-biogeochemical processes for the rest of the twenty-first century.

**Table 1:** Information of the five global climate models (GCMs) used in this study

Model	Resolution (Lon. × Lat.)	Institute
GFDL-ESM2M	720 × 360	NOAA/Geophysical Fluid Dynamics Laboratory
HadGEM2-ES	720 × 360	Met Office Hadley Center
IPSL-CM5A-LR	720 × 360	L’Institute Pierre-Simon Laplace
MIROC-ESM-CHEM	720 × 360	AORI, NIES, and JAMSTEC
NoerESM1-M	720 × 360	Norwegian Climate Center

The output of each GCM was bias corrected and downscaled to the 0.5° × 0.5° spatial resolution

### Analysis of responses of SOC loss to climate warming and SWC

In many previous studies, the SOC loss or decomposition rate had always been found to have a quadratic or exponential relationship with warming or SWC or their interaction [21, 27, 35, 50]. Driven by this trend, we used the Gauss–Newton algorithm to establish the quadratic relationships (model) between the SOC loss and warming or SWC or their interaction. Following previous studies [51–54], we mainly concentrated on the root-zone SWC (i.e., SWC below 10 cm but above 300 cm of the soil depth [55]) and the top 90-cm SOC of the soil profile. There are several reasons why we chose the root-zone SWC and 90-cm SOC in this study. On the one hand, the root-zone SWC plays a vital role in the water-limited Loess Plateau, which is characterized by a thick vadose zone and deep groundwater level, because it is explicitly linked with the vegetation growth and carbon allocation into above- and below-ground biomass [56, 57]. For example, Gao et al. [58] investigated the afforestation effects on deep root-zone SWC (2-8 m) and shallow-layer SOC (0–1.6 m) and their relationships; Feng et al. [59] found there existed significant correlations between root-zone SWC and shallow-layer SOC (< 2 m) by comparing relationships among different soil depths in the Loess Plateau region. On the other hand, global quantification had revealed that ~ 55% of the top 1-m SOC lied below 0.3-m depth and, thus, a top 90-cm SOC could more reasonably represent the SOC content of a specific ecosystem than other depths (e.g., the widely-used 20-cm SOC) [2]. This is especially the case in the Loess Plateau, which has a thick loess deposit with a significant decrease of SOC level along depth. The nonlinear statistical model proposed in this study were:

1 $${ }\Delta C_{t} = a\Delta T^{2}$$

2 $$\Delta {\text{C}}_{swc} = {\text{b}}\Delta SWC^{2} + c\Delta SWC$$

3 $$\Delta C_{{\left( {t + swc} \right)}} = {\text{d}}\Delta T^{2} + {\text{e}}\Delta SWC^{2} + f\Delta SWC$$

where *ΔC*_*t*_, *ΔC*_*swc*_, and *ΔC*_*(t*+*swc)*_ are the SOC changes caused by warming, SWC change, and their interaction, respectively; *ΔT* indicates the warming level (°C) (the air temperature difference between the future conditions and the baseline (1976–2016); *ΔSWC* indicates the change of SWC (mm) (i.e., the average soil water content during the future climate minus the average ones during the historical period (1976–2016)); and a, b, c, d, e, and f represent the fitted coefficients for the statistical models.

We performed model selection in terms of the Akaike information criterion (AIC) and Bayesian information criterion (BIC). A lower AIC or BIC indicates a better fit. Based on the final fitted nonlinear models, we investigated the responses of the SOC loss to climate warming and SWC change under the three RCPs. The impacts of climate change on SOC and SWC were investigated by comparing the differences between the baseline conditions (1976–2016) and future projections (2017–2099) from the model outputs. Besides, we used the nonparametric deviance reduction analysis as an auxiliary [60] to detect the change-point of an ecological indicator along another one. This method estimates the numerical value of a predictor x, resulting an abrupt change in the response variable, y, represented as the cumulative probability of a change-point [61]. Before and after this change-point, the relationship between two environmental indicators (for example, the SWC change and SOC loss in our study) may be different, and thus this point can approximately be the threshold in regulating the responses.

## Results

### Temporal changes in historical and future climate, SWC, and SOC

**Fig. 2:**
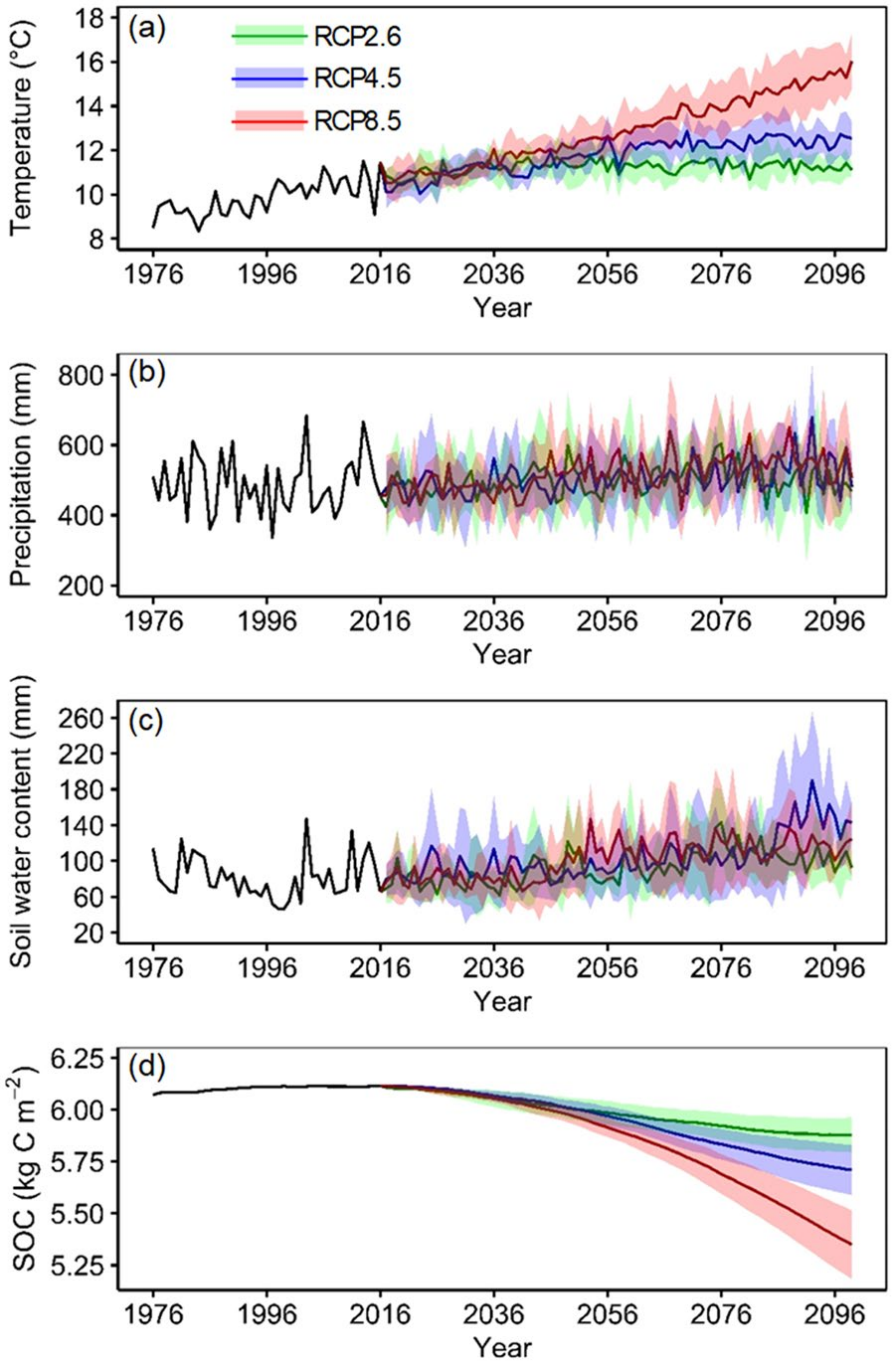
Projected changes in **a** average air temperature, **b** precipitation, **c** soil water content (root zone), and **d** SOC. The shading area (uncertainty range) denotes the ± 1 standard deviation range of model annual averages

The historical and projected changes in climate (air temperature and precipitation), SWC, and SOC under RCP2.6, RCP4.5, and RCP8.5 are shown in Fig. 2. The average air temperature had been increasing during the historical period (1976–2016) and was projected to continuously increase during the future period (2017–2099) under all RCPs, indicating a significant warming trend for the twenty-first century in the JRB. Specifically, the annual mean temperature increased by 1.8 °C from 1976 to 2016, with a rate of 0.46 °C per decade. Although the warming rate would become lower under RCP2.6 (0.02 °C per decade) and RCP4.5 (0.28 °C per decade) from 2017 to 2099, it would reach 0.63 °C per decade under RCP8.5, i.e., 18% of increase compared to the historical period. The annual precipitation showed an upward trend during the historical period (4.2 mm per decade) and was also projected to increase with a rate of 3.8, 7.7, and 12.0 mm per decade during the future period (2017–2099) under RCP2.6, RCP4.5, and RCP8.5, respectively, indicating a wetting trend in the JRB. Compared to the historical condition, the average air temperature and precipitation during the prediction period would increase by 1.4, 1.9, and 3.1 °C and 2.0%, 13.1%, and 6.0% under RCP2.6, 4.5, and 8.5, respectively (Table 2).

**Table 2:** Projected future (2017–2099) changes in climate (temperature (ΔT) and precipitation (ΔP)) and SWC (ΔSWC) and soil organic carbon (ΔSOC) relative to the historical period (1976–2016)

Scenario	ΔT (°C)	ΔP (%)	ΔSWC (%)	ΔSOC (kg C m^−2^ year^−1^)
RCP2.6	1.4	2.0	14.1	− 0.12
RCP4.5	1.9	13.1	27.9	− 0.16
RCP8.5	3.1	6.0	25.4	− 0.26

It was projected that the changes in annual SWC were relatively consistent with the changes in annual precipitation across all climate scenarios (Fig. 2c). The SWC showed a periodic fluctuation during the historical period (1976–2016), while it showed an upward trend during the future period (2017–2099). In comparison with the historical period, the basin-scale annual average SWC would increase by 14.1%, 27.9%, and 25.4% under RCP2.6, RCP4.5, and RCP8.5 by the end of the twenty-first century (Table 2). As can be seen from Fig. 2d, although the SOC increased slightly from 1976–2016, it would decrease across all climate scenarios with the highest depletion rate occurring in RCP8.5 (− 0.09 kg C m^−2^ per decade). As shown in Table 2, when compared to the historical condition, the SOC would decrease by 0.12, 0.16, and 0.26 kg C m^−2^ year^−1^ under RCP2.6, RCP4.5, and RCP8.5, respectively, indicating a potential carbon source under climate warming in the JRB.

### Spatial patterns of SWC and SOC changes under future climate

**Fig. 3:**
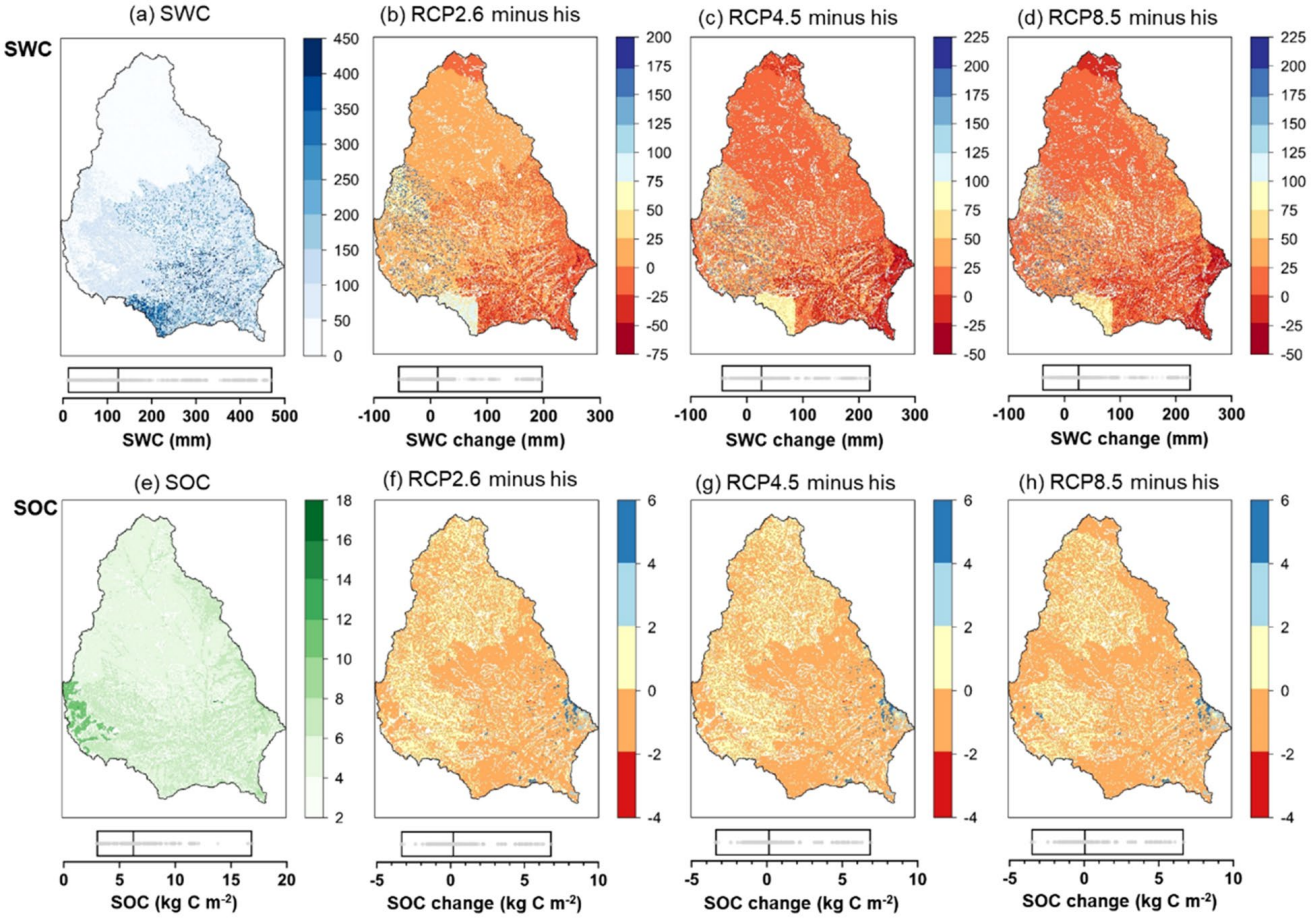
Spatial patterns of SWC (the upper panel, **a**) and SOC (the lower panel, **e**) during the historical period (1976–2016) and projected changes in the future (2017–2099) under RCP2.6 (**b** and **f**), RCP4.5 (**c** and **g**), and RCP8.5 (**d** and **h**). ‘RCP2.6/4.5/8.5 minus his’ indicates the average values of each Hydrologic Response Unit (HRU) during 2017–2099 under RCP2.6, RCP4.5, and RCP8.5 minus the average values during 1976–2016. Boxes indicate the spatial distributions of SWC and SOC with the minimum (left), maximum (right), plus average (the vertical solid lines in boxes) values. Grey dots in boxes indicate the distribution densities of changes of SWC and SOC

Figure 3 shows the spatial patterns of SWC (the upper panel) and SOC (the lower panel) and their changes under RCP2.6, RCP4.5, and RCP8.5. During the historical period, the lower and higher SWC were primarily located in the northern and southern areas, respectively. The annual average SWC showed an increasing gradient from the north to the south with a range of 10 mm to 450 mm (as shown in the side bars) during 1976–2016. Some portions in southern areas and northern margins showed relatively larger decreased magnitudes, while other areas were projected to increase with increased magnitude ranging from 0 to 225 mm. For SOC, the basin average SOC density was 6.2 kg C m^−2^, with higher values in the western margin (Fig. 3). The SOC in most parts of the southern basin were projected to decrease with a decreasing magnitude ranging from − 4 to 0 kg C m^−2^ across the three RCPs (as shown in the side bars). Large areas in the north basin and small portions in the southeastern margin showed an increase in SOC, indicating a potential carbon sink in these areas under the future climate conditions.

### Nonlinear responses of SOC loss to warming and soil water variation

**Fig. 4:**
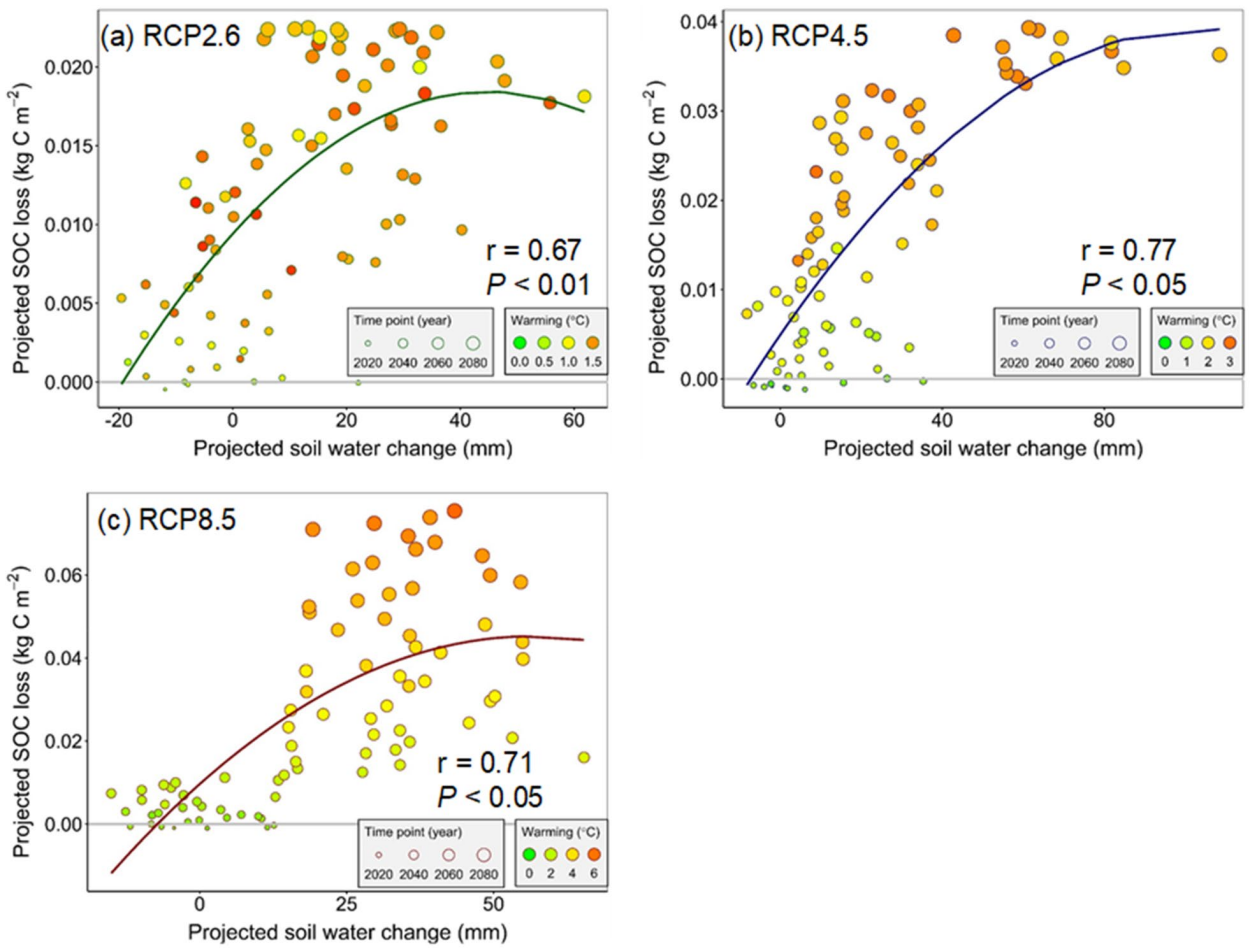
Relationships between changes in SWC and SOC loss under RCP2.6, RCP4.5, and RCP8.5. Bubble size indicates the time point during 2017–2099, and the color shows different warming levels (the air temperature difference between the baseline condition (1976–2016) and future climate conditions (2017–2099))

As stated in "Temporal changes in historical and future climate, SWC, and SOC", SOC was projected to decrease in the future but the decreasing magnitude varied among different climate scenarios. To investigate the nonlinear responses of the projected SOC loss to climate warming and SWC, we used the Gauss–Newton algorithm to establish the nonlinear relationships between the SOC loss and warming (Eq. 1) or SWC (Eq. 2) or their interaction (Eq. 3). When plotting the projected root-zone SWC change against the SOC loss based on Eq. 1 (as shown in Fig. 4), the SWC change and the SOC loss had a significant (*P* < 0.05) quadratic relationship with the correlation coefficients (r) ranging from 0.67 to 0.77. The comparison showed that both the AIC and BIC values in models between the SOC loss and warming or SWC change individually were higher than those between the SOC loss and the interactions between warming and SWC change (as shown in Tables 3 and 4). This phenomenon demonstrated that the best-fit response model should include both warming and SWC change, which could more reasonably explain the SOC loss under future climate conditions. To validate the fitted nonlinear models, we compared the modeling results with those simulated by SWAT-DayCent (Additional file 1: Figure S1). The comparison showed that the best-fit nonlinear model (Eq. 3) could accurately discern the SOC loss across all climate scenarios (r were 0.66, 0.94, and 0.99 under RCP2.6, RCP4.5, and RCP8.5, respectively, *P* < 0.001), and thus we used these nonlinear response models to explain the SOC loss in the JRB.

**Table 3:** Fitted nonlinear models between SOC loss and climate warming and SWC change

Scenario	Model	AIC	BIC
RCP2.6	Δ*C*_*(t*+*swc)*_ = 0.002Δ*T*^2^-0.000002Δ*SWC*^2^ + 0.0001Δ*SWC*	− 611	− 602
RCP4.5	Δ*C*_*(t*+*swc)*_ = 0.003Δ*T*^2^ + 0.0000005Δ*SWC* ^2^ + 0.0002Δ*SWC*	− 646	− 636
RCP8.5	Δ*C*_*(t*+*swc)*_ = 0.004Δ*T*^2^-0.000003Δ*SW*C^2^ + 0.0003Δ*SWC*	− 712	− 702

AIC: Akaike Information Criterion; BIC: Bayesian Information Criterion

**Table 4:** AIC and BIC for the fitted nonlinear models between the SOC loss and warming or SWC

	RCPs	AIC	BIC
SOC loss versus warming alone (quadratic)	RCP2.6	− 575	− 571
	RCP4.5	− 594	− 589
	RCP8.5	− 704	− 699
SOC loss versus SWC alone (quadratic)	RCP2.6	− 530	− 523
	RCP4.5	− 541	− 534
	RCP8.5	− 429	− 427
SOC loss versus warming and SWC (exponential combined with quadratic)	RCP2.6	− 529	− 520
	RCP4.5	− 574	− 565
	RCP8.5	− 569	− 559

AIC: Akaike Information Criterion; BIC: Bayesian Information Criterion

**Fig. 5:**
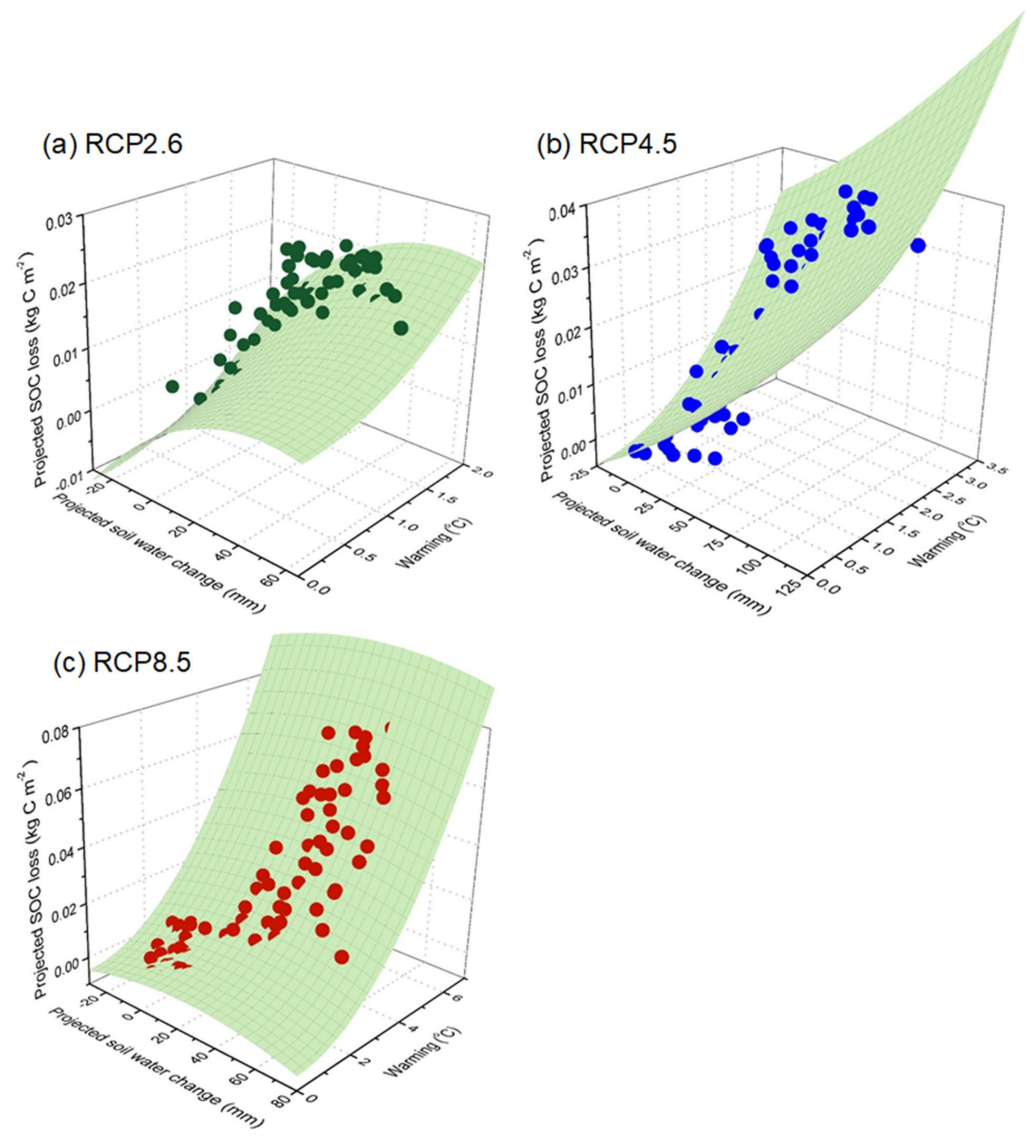
Response surfaces of the relationships between root-zone SWC and climate warming and SOC loss under RCP2.6, RCP4.5, and RCP8.5. Observed values (the green, blue, and red dots) are the means of projected SOC loss from five GCMs, and the modeled values (the green surfaces) are predictions from the fitted nonlinear models

Based on the final fitted nonlinear model, we generated the response surfaces (Fig. 5). As shown in Fig. 5, the hump-shaped response surfaces were found in both RCP2.6 and RCP8.5, whereas this was not evident in the RCP4.5. A clear ridge (i.e., the threshold of changing SWC) was observed under both RCP2.6 and RCP8.5 (Fig. 4). The changing threshold of SWC was 25 and 50 mm under RCP2.6 and RCP8.5, respectively. Under both RCP2.6 and RCP8.5, when the changes in SWC were lower than the thresholds, warming would accelerate the SOC loss from soil, whereas the soil water could alleviate the warming-induced SOC loss when it was higher than this threshold. This phenomenon demonstrated the key role of SWC in affecting the SOC loss under a warming climate. However, this water mediation of warming effects was not detected in RCP4.5, where the SOC loss increased following the warming and SWC gradient (Fig. 5b). It was found that the SOC loss increased with increasing SWC but kept relatively stable after reaching a certain level (a quadratic relationship with a downward opening) (Fig. 4b), where the SOC loss was almost unchanged across gradients of warming and SWC. To explain this phenomenon, we used nonparametric deviance reduction analysis to detect the SWC change-point along SOC loss and found that the relationship change occurred at 41 mm of projected SWC change (Fig. 4b), indicating that the SWC could still mediate the response of SOC loss to warming across the full range of warming under RCP4.5 when considering SWC alone. However, this water mediation diminished when interacting with climate warming and the combined effects of SWC and warming on SOC loss would change to be positive.

## Discussion

### SOC loss caused by warming

**Fig. 6:**
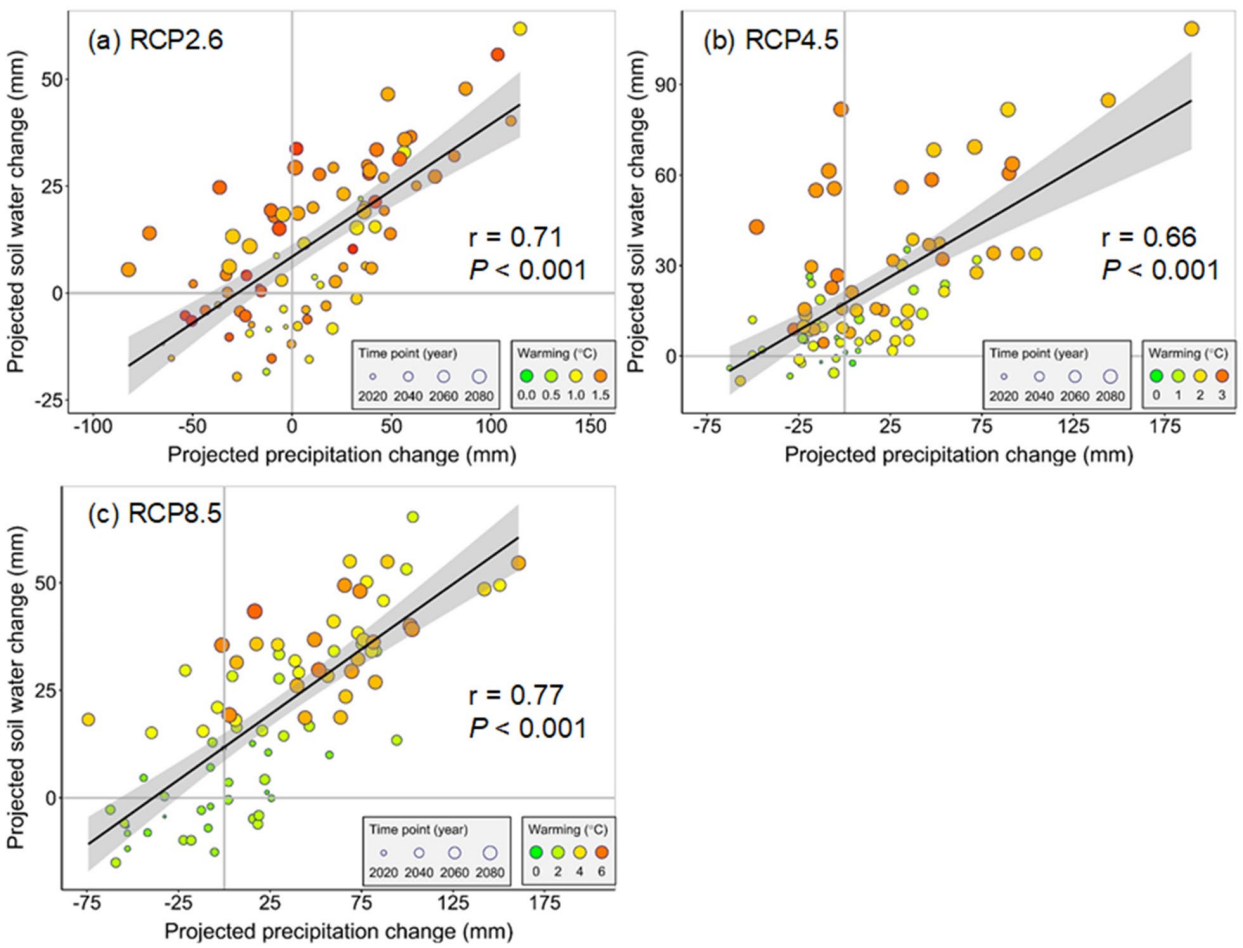
Relationships between changes in precipitation and SWC under **a** RCP2.6, **b** RCP4.5, and **c** RCP8.5. Bubble size indicates the time point during 2017–2099, and the color shows different warming levels

Predicting the spatiotemporal changes in SOC is of great importance, as it is explicitly linked with the soil functions and many ecosystem services. Our study predicted that SOC during the future period would become significantly lower across all climate scenarios. This phenomenon demonstrated a potential carbon source of the JRB in the twenty-first century, which has also been confirmed by previous studies [11, 62, 63]. The significant SOC depletion can be attributed to the warmer climate in the future, despite the increased SWC induced by an increased precipitation (Fig. 6) [23, 64]. The warming effects on the SOC depletion can be explained by several candidate mechanisms. On the one hand, elevated air temperature can induce an increase in soil temperature, which would activate the microbial activities and accelerate the SOC decomposition rate [65]. On the other hand, both the meta-analysis and experimental results, from past studies, have indicated that the plant photosynthesis is more sensitive to the warming/drought than respiration [31, 66, 67]. A global-scale study has also revealed that a warmer year (less water availability) was always associated with faster CO_2_ growth, demonstrating the critical role of warming in the SOC decomposition [68]. Some quantitative studies have also assessed the response of SOC to climate warming, which were in line with our study [69]. For example, an expected increase in air temperature of 3.3 °C would cause an SOC loss of 11–16% over the Europe, and an average increase in surface air temperature of 1 °C would cause a net loss of 5% of the SOC pool globally. In brief, our analysis demonstrated that anthropogenic-induced future climate warming alone would always result in SOC loss across the full range of SWC.

### Water-mediated warming effects on SOC

Soil water content (water availability) plays a crucial role in determining the response of the SOC loss to climate warming through affecting the soil respiration and plant productivity directly [70–72]. Many previous experimental studies have revealed a quadratic relationship between the soil water content and soil respiration (SOC loss), which were in line with our findings in the JRB. For example, Jassal et al. [70] found that there existed a quadratic relationship between the basal respiration and SWC in an old temperature Douglas-fir stand. In a constructed old-field grassland, Wan et al. [36] also reported a second-order polynomial function between SWC and soil respiration. These studies support our findings that the SWC would exert different effects on SOC under different contents—a quadratic relationship between the predicted SOC loss and SWC (Fig. 4). When plotting the combined effects of warming and SWC change against the predicted SOC loss, we found hump-shape response surfaces under RCP2.6 and RCP8.5, which implied that the warming effects on SOC (warming-induce SOC loss) were meditated by the SWC changes. Under the warming climate in both RCP2.6 and RCP8.5, the SWC would stimulate the SOC loss when its change was below the threshold (the ridge of the hump-shaped surface) (a drier or slightly wetter trend compared to the baseline condition), while the increased root-zone SWC would alleviate the warming-induced SOC loss when its positive change was above the threshold (a relatively large wetter condition) (Fig. 5). The threshold became higher under RCP8.5 (high warming level) than that under RCP2.6 (low warming level). In general, under adequate water availability, warming can consistently promote plant growth and enhance C sequestration [27, 73], while the warming-induced droughts can also limit both C uptake and soil respiration under the water deficit conditions [74, 75]. These two mechanisms might be the reasons that the magnitude of SOC loss increased first and then decreased with increasing the SWC under the warming climate in our estimated response surfaces. The findings highlighted that the positive effects of warming on SOC loss could be partly mediated by SWC, which may lead to variable C-climate feedbacks under different SWC conditions in the JRB. Our findings may also have implications for the managements of terrestrial ecosystems under the future warmer climate. Ecosystems in dry regions (lower SWC) most likely act as carbon sources under future warmer climate conditions and thus cause a positive feedback to climate warming, while ecosystems in wet regions (higher SWC) possibly generate a negative feedback. The policy makers should seek different management measures in ecosystems with different SWC under the future warmer climate. Actually, this mechanism derived in our study was generally supported by the global-scale study that warming stimulates carbon release in low-precipitation regions but enhances the carbon uptake in high-precipitation areas [27]. Thus, some studies held that there existed a threshold of precipitation that regulated the net carbon exchange at continental scales [34].

It was worth noting that this water mediation was not found in RCP4.5, which might be attributed to the higher inter-annual variability (IAV) of SWC in RCP4.5 compared to that in RCP2.6 and RCP8.5 (Additional file 1: Figure S2) and the IAV-induced inconsistent change of SOC loss (Additional file 1: Figure S3). A higher IAV (less stable) of SWC in this water-limited loess watershed would exert a persistent stimulation for the microbial activities, leading to continuous decomposition of SOC under the climate warming [76, 77]. According to the research implemented in semiarid steppes by Rey et al. [76], a sudden change in soil moisture caused by higher IAV of precipitation would contribute to about 65 ~ 80% of total carbon losses at different vegetation covers. An experimental study conducted in the Loess Plateau, which is similar to our study area, has proved that the higher variability of SWC caused by pulsed rainfall could significantly stimulate the C loss from soil and explain a large portion of the variation in soil respiration (above 50%) [78]. Thus, the combined warming and SWC change and IAV contributed to a persistent SOC loss in the JRB, which raised a caution to consider the variability of water availability (including both precipitation and SWC) when investigating the C-climate feedbacks.

### Limitations and uncertainties

In our study, we used the quadratic functions to analyze the relationships between the SOC loss and warming or SWC or their interaction, which may simplify the mechanism of the responses of the C cycle to warming and hydrological cycle. In fact, many previous experimental studies have used a variety of response functions, such as linear [12], quadratic [31, 79], and exponential [80], to depict the carbon cycle responses to warming and SWC. For example, Quan et al. [27] combined a quadratic function between NEP (net ecosystem productivity) and SWC and an exponential function between carbon cycles and climate warming to generate a continuous model to discern the water scaling pattern of warming effects on the carbon cycle. In the present study, we also tried the exponential function between climate warming and the SOC loss by reproducing their approach [27] but achieved only poor performance (Table 4). This phenomenon demonstrated that the quadratic model used in our study was more reasonable to represent the C response to climate warming and SWC change in the JRB.

Uncertainties involved in this study can be primarily attributed to the GCMs and the hydrological modeling approach. Considering the sophisticated climate system and the potential uncertainties of the GCM outputs, we used an ensemble of five GCMs to drive the hydro-biogeochemical model and could observe a certain level of uncertainties in the predicted water and carbon components (see the uncertainty bands in Fig. 2). Though the model verification showed that SWAT-DayCent could accurately simulate the water and carbon cycles in the JRB, it was inevitable that bias would occur due to the multiple influencing factors of remotely-sensed NPP, such as meteorological conditions (clouds and aerosols), vegetation conditions, and sensors. In addition, we assumed that the land use information was constant in the simulations, which might have led to an overestimation or underestimation of the water and carbon components. We will consider the dynamic land use information in the simulations using SWAT-DayCent and other process-based models in future studies. It is worth noting that we only investigated SOC change and its responses to climate warming and SWC change in this study and did not consider other driving factors (e.g., the plant/animal litter input, the soil respiration loss, and nutrient stimulation). We noted these factors may play important roles in driving SOC changes in the JRB during our study period, leading to uncertainties in the analysis. Future studies could consider more factors in driving SOC change to gain a more comprehensive understanding of how carbon cycle responses to the climate change under a more complex environment.

## Conclusions

In this study, we quantitatively investigated the spatiotemporal changes in soil organic carbon (SOC) and its responses to both climate warming and soil water content (SWC) in the Jinghe River Basin (JRB) in China, by combining the Global Circulation Model (GCM) output and a coupled SWAT-DayCent model. The future climate projections showed that the air temperature would increase significantly across all the three scenarios considered (RCP2.6, RCP4.5, and RCP8.5), and the precipitation would increase by 2.0–13.1% by the end of the twenty-first century, indicating a warmer and wetter climate during the twenty-first century in the JRB. The SWC was projected to increase by 14.1–27.9% owing to the increased precipitation, while the SOC would significantly decrease during the future period, with varying degrees among the different climate scenarios. Through analyzing the relationships between the projected SOC loss and climate warming, SWC change, and their interaction, we showed that there existed a changing SWC threshold which could mediate the warming-induced SOC loss. When the SWC change was lower than the threshold, a higher SWC would accelerate the SOC loss; when the SWC change was higher than the threshold, a higher SWC would depress the SOC loss. This water mediation pattern would help the watershed managers make effective and efficient policies and measures to enhance the C sequestration in a warming climate.

## Supplementary Information

**Additional file 1.** Additional figures.

## Data Availability

The data and materials are available upon reasonable request.

## Abbreviations

JRB: Jinghe River Basin
SWAT: Soil and Water Assessment Tool
SOC: Soil Organic Carbon
SWC: Soil Water Content
NPP: Net Primary Productivity
ET: Evapotranspiration
GCM: Global Climate Model
RCP: Representative Concentration Pathway
ISI-MIP: Inter-Sectoral Impact Model Inter-comparison Project
CMIP5: Coupled Model Inter-comparison Project Phase 5
DEM: Digital Elevation Model
CRU: Climatic Research Unit
SRTM: Shuttle Radar Topography Mission
CMA: China Meteorological Administration
AIC: Akaike Information Criterion
BIC: Bayesian Information Criterion
NSE: Nash–Sutcliffe Efficiency
