# Supplementary material for: Projected soil organic carbon loss in response to climate warming and soil water content in a loess watershed
Source: Carbon Balance Manag. 2021 Aug 16;16:24. doi: 10.1186/s13021-021-00187-2 (PMC8369727; doi:10.1186/s13021-021-00187-2)
Supplement: Supplementary file 1 — Additional file 1. Additional figures. [file 13021_2021_187_MOESM1_ESM.docx]

**Figure S1.** Relationships between the predicted SOC loss using SWAT-DayCent and the modeled SOC loss using the nonlinear fitted model (equation 3). The shading area denotes the 95% confidence interval.

**Figure S2**. Comparison of the SWC change (ΔSWC), inter-annual variability (IAV), and coefficient of variation (CV) under RCP2.6, RCP4.5, and RCP8.5.


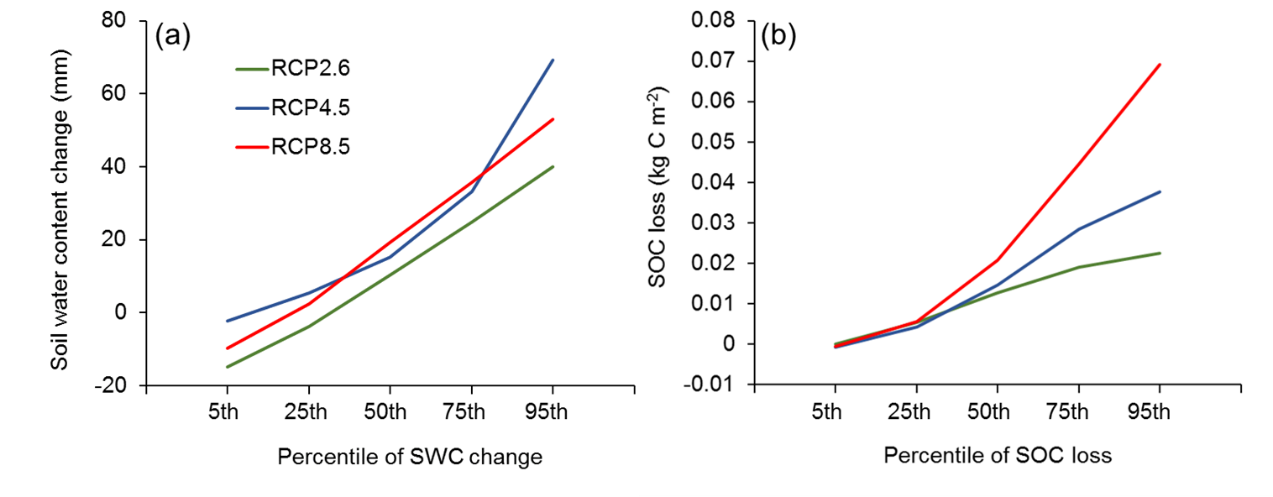


**Figure S3.** Changes in SWC and SOC loss along their percentiles within the study period (2017-2099).
